# Coral Restoration in the Omics Era: Development of Point‐of‐Care Tools for Monitoring Disease, Reproduction, and Thermal Stress

**DOI:** 10.1002/bies.70007

**Published:** 2025-04-26

**Authors:** Erin E. Chille, Timothy G. Stephens, Shrinivas Nandi, Haoyu Jiang, Michael J. Gerdes, Olivia M. Williamson, Alexander Neufeld, Phanor Montoya‐Maya, Debashish Bhattacharya

**Affiliations:** ^1^ Department of Biochemistry and Microbiology Rutgers University New Brunswick New Jersey USA; ^2^ CapitalCorals INC Albany New York USA; ^3^ Revive & Restore Sausalito California USA; ^4^ Coral Restoration Foundation™ Tavernier Florida USA

**Keywords:** biomarkers, coral health and disease, coral reefs, coral reproduction, molecular diagnostics, restoration, thermal stress

## Abstract

Coral reef degradation has captured global attention from governments, conservationists, and researchers, who are making concerted efforts to develop sustainable solutions to support reef resilience in the face of environmental degradation. The goal is to empower local community efforts for effective marine resource management. However, one of the major barriers to coral conservation is the lack of timely and affordable population‐level health data, which can delay effective management responses. Although progress has been made in understanding the molecular basis of coral health outcomes, more translational work is needed to develop cost‐effective, point‐of‐care (POC) diagnostic tools for real‐time monitoring. This review assesses the current state of translational omics‐based research for coral health monitoring, focusing on highlighting key gaps and actionable next steps to guide the implementation of effective, field‐ready tools for monitoring coral disease, reproduction, and thermal stress. These advancements can be used to advance urgent conservation needs and promote reef management by local communities.

## Graphical abstract

Shown are different multi‐omics approaches that are used to identify potential biomarkers of coral health and disease. The integration of these data streams, using cutting‐edge molecular diagnostic technologies, including colorimetric dipsticks, lateral flow assays, and colorimetric LAMP (loop‐mediated isothermal amplification), holds the promise to address urgent conservation needs and promote reef management by local communities. Figure created using Biorender.

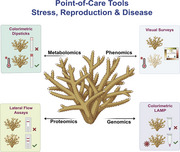

**Abbreviations**
CBASS: coral bleaching automated stress system
CMSA: coral mass spawning asynchrony
DART‐MS: direct analysis in real‐time mass spectrometry
POC: point‐of‐care

## 1 Introduction

**FIGURE 1:**
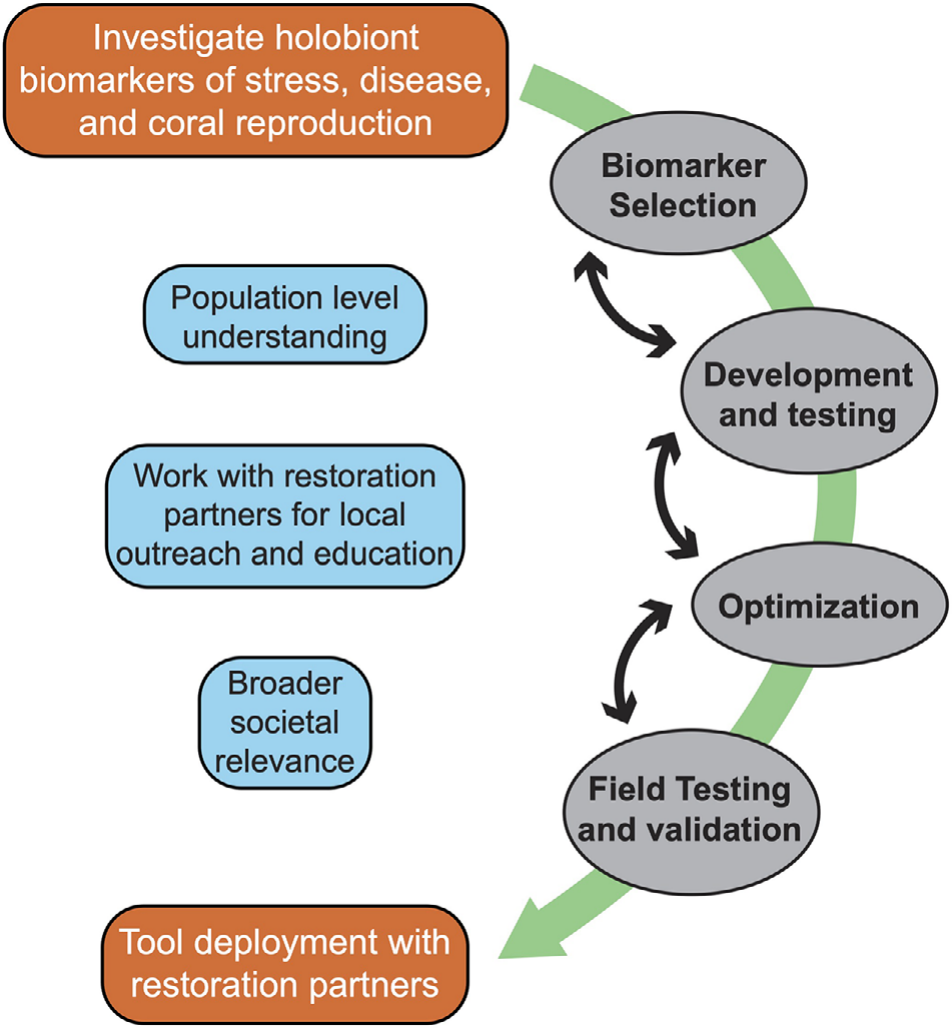
A stepwise, iterative approach to coral toolkit development and deployment using multi‐omics data that is organized around the core goals of biomarker selection, development, optimization, and field‐testing. Achieving a population‐level understanding of coral health, followed by work with restoration partners and outreach to the public, are key elements to inform tool development and impact.

The accelerating decline of coral reefs globally has spurred the rapid growth of coral restoration programs [1]. Most of these efforts seek to mitigate the loss of coral assemblages by collecting gametes or fragments from wild, reef‐building coral colonies, growing them out in in situ and/or ex situ nursery systems, and then outplanting them back onto degraded sites. Over the past four decades, reef restoration projects have evolved from pilot studies focused on testing feasibility, evaluating methods, and assessing coral survivorship to multi‐year restoration and intervention efforts over large regions [2]. Coral conservation continues to expand in size, scope, and geographic reach as its integral role in reef management is increasingly recognized [3]. However, the work of the coral restoration community and the health of reefs remain vulnerable to global climate change [4]. In addition, coral reefs largely exist in nation‐states that are disproportionately impacted by environmental degradation. These nations contribute the least to global emissions yet are the most financially and culturally at risk and have the fewest resources available to preserve local ecosystems [5]. Thus, there is a growing need for coral monitoring solutions that: (1) are low‐cost, (2) can be deployed easily, and (3) rapidly return actionable insights into how restored and wild corals are responding to warming oceans, harmful algal blooms, turbidity, water pollution, and emerging diseases [6]. Such tools would allow coral restoration practitioners to make more informed, proactive decisions about the techniques and logistics surrounding their specific restoration program during episodic threats to corals. Here, we briefly review the current state of coral health monitoring using cutting‐edge multi‐omics methods. These insights lead us to suggest an adaptive framework for toolkit implementation that can be used to monitor coral health and reproduction and iterated upon through deployment and outreach (Figure 1).

As for human populations, health outcomes for coral assemblages depend on the interplay between standing genetic variation and how this interacts with environmental and other risk factors. Environmental risks (e.g., thermal stress, eutrophication, overfishing of herbivorous fishes, pollution, and disease) are of primary concern for restoration practitioners [7]. Coral assemblages exist along a spectrum of environmental risks. Whereas some reefs thrive in relatively pristine waters, such as those in the biodiverse Coral Triangle [8], many others, like those in the Caribbean, face continuous stress from warming waters reef management, disease, and local environmental pressures [9]. Monitoring needs may differ for reef assemblages on either end of the spectrum, with healthy reefs primarily targeted for long‐term observation (e.g., “checkups”) and more disturbed regions targeted for risk/impact assessment.

Global collaborations and growing interest in coral conservation have advanced coral health monitoring beyond small‐scale visual surveys toward quick and effective screening of large reef areas [10, 11, 12]. Increasingly, large‐scale aerial and submersible surveys and portable systems to assay coral resilience on the regional scale [13] are being adopted for coral monitoring. The Coral Bleaching Automated Stress System (CBASS) is an example of a low‐cost and field‐portable platform that has been widely adopted for coral health diagnostics. CBASS is an experimental system that, using standardized temperature stress profiles, rapidly assesses thermal thresholds using standard bleaching metrics such as chlorophyll‐*a* content, symbiont cell density, and photosynthetic efficiency (*F_v_/F_m_*) [14]. Methods such as color score [15], the Community In situ Metabolism device (CISME Instruments, LLC) [16], the grafting‐based genotypic diversity assay [17], and the Fluorescence Imaging System (FluorIS) for recruitment detection [18] provide other examples of lower‐cost, low‐barrier, portable systems to assay population‐ or community‐level metrics related to coral health. Although these methods are effective for reef monitoring, additional data on coral health and resilience, such as those described below for coral disease, mass spawning, and thermal stress, could add important, actionable insights for reef managers. Using integrated multi‐omics data including transcriptomics, proteomics, and metabolomics, rapid point‐of‐care (POC) tests can be developed using **biomarkers** (see **Box**) of coral health and adaptive capacity. Here, we highlight key areas of coral monitoring and how they can be enhanced using molecular diagnostic technologies developed for human health and disease monitoring, which can be readily translated for coral conservation. Below, we discuss strategies for achieving this aim, but first provide a brief primer on coral biology.

### 1.1 The Coral Holobiont

The partnership between cnidarian hosts and single‐celled dinoflagellate (algal) endosymbionts in the family Symbiodiniaceae “powers” tropical coral reef ecosystems through the provision of photosynthates and other nutrients [19]. However, under thermal stress, the nutritional exchange between the coral host and the endosymbiont is disturbed, which can lead to the expulsion of algae from coral tissues (i.e., “bleaching”) [20]. In this state, the coral host is susceptible to threats such as disease or predation [21], and may die if the symbiosis is not reestablished [22]. Beyond thermal stress, other environmental impacts, including sedimentation, eutrophication, and overfishing of herbivorous fishes also impact coral health [6]. Because marine heatwaves are one of the leading causes of global coral mortality [23], biomarker discovery to aid coral monitoring efforts has primarily focused on developing a library of nucleic acid markers indicative of stress, adaptation, or acclimation to warmer temperatures [24]. However, many of these putative biomarkers have yet to be assessed for their ability to diagnose other, or combined stressors. In addition, reproductive challenges, such as mass spawning asynchrony, which limits population recovery after mass mortality events [25] (discussed under **Section** **3.2**) and disease outbreaks [26] (discussed under **Section** **3.1**) pose significant challenges for coral restoration efforts. Additional work is needed to identify, validate, and integrate biomarkers that encompass the broad range of often co‐occurring health challenges corals face into field‐ready tools that can improve coral health monitoring.

Coral health and adaptive capacity not only depend on the host and its symbiotic algae (Symbiodiniaceae) but also on a complex association of microorganisms that make up the **coral holobiont** (see **Box**), including protists, fungi, viruses, and prokaryotes [27]. The range of responses to stress varies greatly both within and between coral populations and species due to these differences in natural history, geographic origin, algal symbiont composition, microbiome composition, developmental stage, and genotype [28, 29, 30, 31]. A key example of this is transmission strategy. Although most broadcast‐spawning coral species acquire symbionts each generation via horizontal transmission from the local environment, only about 25% of broadcast spawners vertically inherit their symbionts [32, 33]. The selective forces driving each of these strategies likely reflect environmental pressures either to rapidly acclimate to variable conditions by acquiring locally adapted algal lineages each generation (via horizontal transmission), or to maintain longer‐term relationships with the symbionts in more stable habitats (via vertical transmission) [34]. Due to the diversity of selective forces and biotic interactions contributing to coral holobiont biology, understanding the drivers of variation in coral stress resilience and the genomic basis for adaptive capacity remain challenging. Despite this limitation, multi‐omics methods have identified several putative markers of stress and adaptation in the coral holobiont that have yet to be validated and integrated into field‐deployable tools [24]. Below, we describe the current state of molecular biomarker‐based diagnostic tools for coral health monitoring, focusing on metabolomics and proteomics, which are less well‐studied than transcriptomic responses. We also provide an overview of relevant applications for biomarker‐based approaches, highlighting existing and emerging tools that can be incorporated into a coral health monitoring toolkit.

**Box**

- **Coral holobiont**: The coral host and its associated microbial community, including Symbiodiniaceae (algal symbionts), bacterial, and archaeal cells, viruses, fungi, and other microorganisms. The interactions within the holobiont influence coral health and resilience.
- **Biomarkers**: Measurable biological molecules (e.g., genes, transcripts, proteins, or metabolites) that may be predictive or diagnostic of the health status or adaptive capacity of a coral colony.
- ***In hospite***: The study of Symbiodiniaceae (algal symbionts) and other microbial partners residing within coral tissues.
- ***Ex hospite***: The study of Symbiodiniaceae and other microbial partners existing outside of coral tissues, such as in the surrounding water column, sediments, or in culture.
- **Dark genes**: Genes of unknown function that are taxonomically restricted to reef‐building corals in the order Scleractinia. These genes are often differentially expressed under stress and likely evolved in response to specific evolutionary constraints faced by the coral host, making them useful targets for biomarker discovery.
- **Stony Coral Tissue Loss Disease (SCTLD)**: A highly virulent and lethal coral disease that causes rapid tissue loss in stony corals.
- **Coral Mass Spawning Asynchrony (CMSA)**: The disruption of synchronized coral spawning, often caused by warming oceans, light pollution, or other environmental or natural stressors. CMSA reduces fertilization success, limits genetic mixing, and threatens the recovery of coral populations following disturbances.

## 2 Development of Biomarker‐Based Tools for Coral Health Monitoring

### 2.1 The Current State of Biomarker‐Based Diagnostics for Coral Health Monitoring

Biomarker‐enabled coral health diagnostics is still in its infancy. Despite recent interest in POC tests to aid coral restoration efforts, tool development has largely stalled at the biomarker discovery phase, primarily due to a lack of infrastructure, as well as logistical and financial constraints [24]. Many biomarkers, particularly RNA, metabolites, and proteins, are highly labile at room temperature, requiring preservation at −80°C to maintain sample integrity. However, standard preservation methods, such as liquid nitrogen, dry ice, and ultra‐low freezers are expensive and often unavailable in remote field locations. Recent advancements in biomolecule preservation have led to the commercial availability of room temperature stabilization products, including DNA/RNA Shield (Zymo Research), RNAlater (ThermoFisher Scientific), and OMNImet·GUT (DNA Genotek). These reagents enable the preservation of nucleic acids, proteins, and metabolites, respectively, for limited periods at room temperature and extended storage at −20°C, enabling the safe collection and shipping of samples from coral reefs in regions with limited cold‐chain storage. Beyond sample preservation, financial and logistical barriers remain a significant challenge for biomarker research. The laboratory equipment and resources needed for molecular analyses, such as mass spectrometry and high‐throughput sequencing, are often unavailable or cost‐prohibitive in many tropical regions where coral reefs are located. As a result, global collaborations, knowledge‐sharing networks, and partnerships with well‐funded institutions are crucial for advancing biomarker validation and implementation. In addition, funding for research beyond biomarker discovery is limited to “WEIRD” (Western, Educated, Industrialized, Rich, and Democratic) nations, is highly competitive, and often requires business collaboration. Expanding funding opportunities through interdisciplinary partnerships, public‐private collaborations, and conservation technology initiatives will be essential to bridge this gap and translate biomarker research into field‐ready tools.

Due to rapid developments in next‐generation sequencing technology, biomarker development has focused primarily on the discovery of genomic and transcriptomic biomarkers for coral health and resilience [35]. Nucleic acid biomarkers have primarily been measured using PCR‐based assays, which require heavy, expensive equipment and a sterile lab setting. As such, this powerful technique has yet to be widely implemented for coral health monitoring in the field. However, this may change with other approaches, such as the recently developed nanoball‐based microfluidics device for the detection of loop‐mediated isothermal amplification (LAMP) products [36], which can enable the sensitive and automated quantification of DNA and RNA biomarkers. This approach would allow the development of rapid field deployable tools for transcript or pathogen (DNA) biomarker detection.

The recent adoption of high‐throughput mass spectrometry techniques in clinical settings is paving the way for metabolomic and proteomic data‐based assays in marine research [37]. In terms of diagnostic technologies, assays for the rapid and inexpensive measurement of metabolite and protein biomarkers from central metabolic pathways have advanced faster than assays to detect nucleic acids due to their large‐scale implementation (and validation) in clinical settings. Existing human POC platforms using metabolite and protein biomarkers, such as antibody‐based lateral flow assays (e.g., pregnancy and COVID‐19 detection kits) and colorimetric chemistry assays (e.g., human urinalysis dipsticks [see below] and diabetic glucose meters), are “low hanging fruit” that can be adapted for coral health monitoring. However, additional research is needed to curate a library of validated protein or metabolite biomarkers of coral health that will be ready to integrate into existing POC testing platforms.

### 2.2 Considerations for Biomarker Discovery and Validation

To effectively adapt existing human POC assays for coral health monitoring, the variation within and among coral holobionts must be considered. Finding conserved markers across all of Scleractinia is likely to be challenging due to the vast evolutionary divergence within this lineage; the estimated split time of stony corals is ∼240 million years [38, 39], as compared to the human lineage, which diverged from New World Monkeys ∼30–35 million years ago [40]. To account for this diversity, biomarker discovery should focus on developing both (1) a validated set of core metabolic or protein markers that are conserved across all Scleractinia or Metazoa, and (2) various sets of biomarkers that target particular populations with shared biology and risk factors (e.g., *Acropora cervicornis* and *Acropora palmata* in the Caribbean region).

Validation for conserved biomarkers across Scleractinia must include testing for species‐ and population‐level needs for biomarker sensitivity. For example, Seveso et al. [41] showed differences in the modulation of Heat Shock Protein 60 (HSP60) under bleaching in different species. Although all species exhibited an initial increase in HSP60 levels followed by a decline correlated with bleaching sensitivity, the magnitude and timing of these responses varied between species. Although HSP60 could serve as a potential marker for stress resilience, it serves as a prime example of the need to delineate detection limits and sensitivity of POC devices to ensure reliable diagnostics across target species. Validation of conserved biomarker responses across species will be particularly salient for reproductive biomarker development due to the vast diversity of reproductive strategies (e.g., dioecious, monoecious, sex switching, pseudo‐gynodioecious) in coral lineages [33]. Therefore, validation of core markers is likely to remain a major hurdle for the development of broadly‐applicable POC tool development.

Standardization also remains a major hurdle for designing biomarkers that target specific populations, because biomarkers effective in one coral population or geographic region may not be universally applicable across the species. First, experiments must consider the genetic structure of the population. Coral fragments used in experiments should reflect the genetic diversity of the target population to account for the genotype‐driven variation in endophenotype (e.g., gene expression, metabolome, proteome), which can often be stronger than stress‐driven variation in corals and other organisms [42, 43, 44]. Another consideration is that corals experience multiple, often co‐occurring stressors, requiring biomarkers to be validated for their specificity in diagnosing or predicting the target condition.

In terms of biomarker discovery, current coral biomarkers have primarily targeted host biology, with few addressing the algal and prokaryotic members of the holobiont. Symbionts have facultative lifestyles, whereby they live both as free‐living cells and within the coral host [45] and have maintained this “dual lifestyle” for the >200‐million‐years that corals have existed [32, 46]. The large Symbiodiniaceae gene repertoire (>30K) [47], makes it clear that holobiont interactions comprise complex models of study under both stable and stressful environmental conditions and are key to understanding and predicting coral responses to stress. Therefore, conservation efforts should also address symbiont biology and biochemistry, both ***in hospite*** and ***ex hospite*** (see **Box**).

Following the model for POC test development in humans, large‐scale field studies that incorporate a variety of environmental risk factors and a taxonomically diverse species pool are needed to validate diagnostic biomarkers. Likewise, long‐term longitudinal studies that also account for taxonomic and environmental diversity are needed to validate predictive biomarkers of adaptive capacity and resilience. In summary, as we advance our discovery and validation of coral health biomarkers, experimental design must incorporate the diversity of the entire holobiont to develop effective universal field‐applicable tools that can be implemented across systems and species.

## 3 Applications for Biomarker‐Based Tools

Below, we summarize three major applications for biomarker‐based coral monitoring tools and the current state of tool development associated with each. We focus on metabolite and protein biomarkers because, as discussed above, the frameworks to integrate these biomarkers into testing platforms already exist for human diagnostics and are “low‐hanging fruit” for coral health monitoring.

### 3.1 Coral Disease Diagnostics

Coral disease is one of the leading causes of global coral mortality [7]. As such, there is a growing need to monitor its spread in wild populations [48] and identify asymptomatic carriers to prevent transmission in coral nurseries [49]. However, diagnosis of coral diseases has historically been challenging due to a lack of reliable tools to identify the disease‐causing agent(s) impacting the coral (e.g., stony coral tissue loss disease [SCTLD]) [48], and molecular differentiation between similar pathologies. To date, diseases have largely been identified based on the color and pattern of the affected area (e.g., White, Yellow, Black) [50, 51]. It is currently unknown if the various phenotypic presentations of each disease are from the same or different causative agent(s). DNA approaches that are often successful in other non‐model organisms [52] are now being coupled with phenotypic evidence of disease to determine the causative agent(s) [48]. However, DNA‐based disease diagnosis presents unique challenges in corals. First, removing diseased tissue for analysis can harm the colony at the location of the disease lesion. Second, because coral tissue samples also contain symbiotic dinoflagellates, bacteria, and viruses, DNA approaches must be designed to account for all components and permutations of the holobiont to determine if a disease‐causing agent is present. This is complicated, especially given that some diseases seem to involve a consistent consortium of otherwise non‐pathogenic prokaryotes [53, 54, 55]. These microbial components reflect location, season, and coral type (i.e., corals have unique symbiont partners and demonstrate seasonal fluctuations) [27]. Due to the heterogeneity of samples, it continues to be extremely difficult to develop a reliable diagnostic platform. Therefore, anything but canonical disease presentations are difficult, if not impossible, to diagnose using standard visual phenotyping and DNA approaches [56]. Progress in this area will be significantly aided by the development of more accurate methods for disease classification.

**FIGURE 2:**
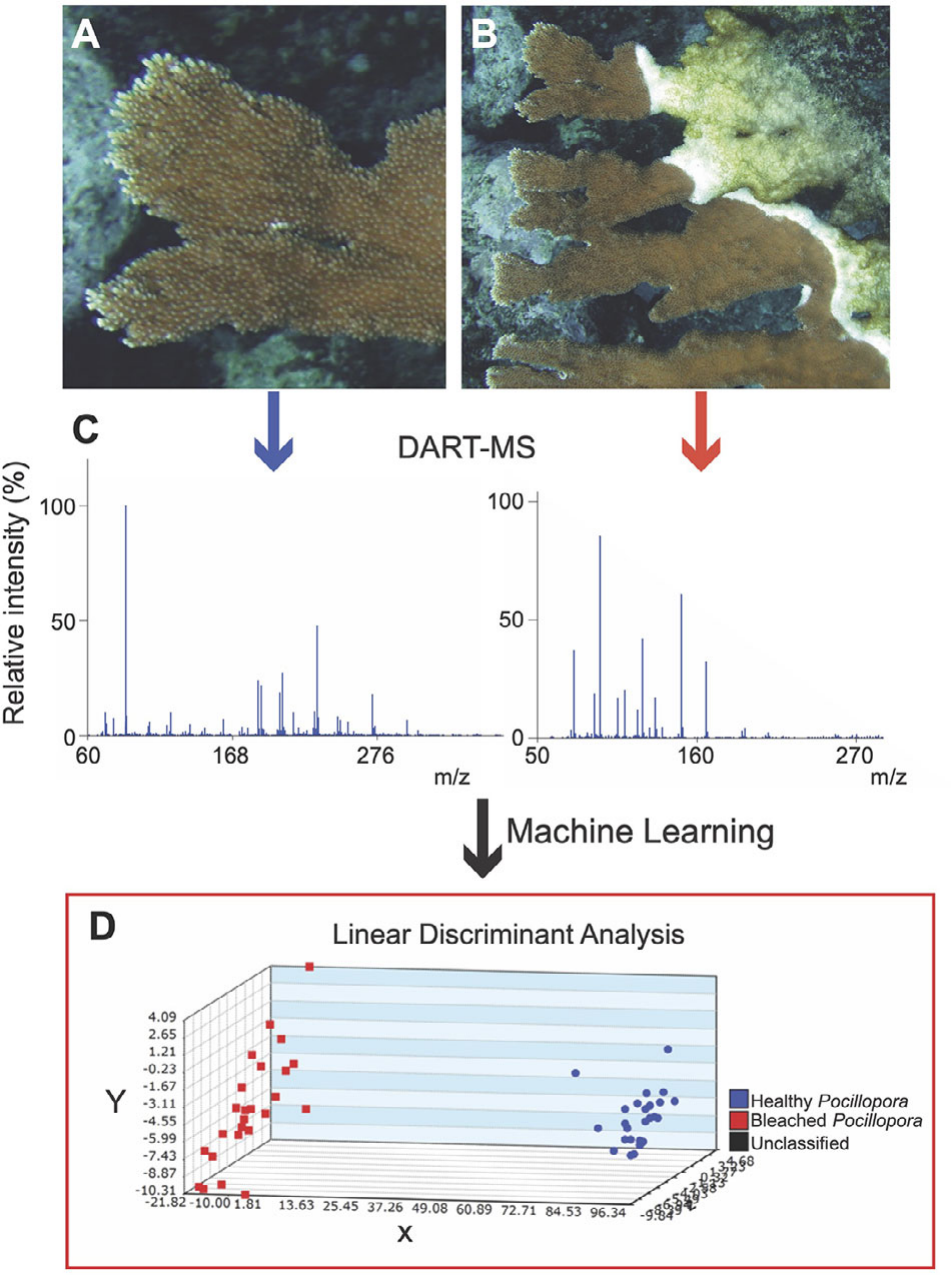
Sampling and analysis of coral disease. (A) Healthy *Acropora palmata* and (B) White band disease in *A. palmata*. (C) DART‐MS profiles are unique for disease versus healthy tissue as shown in (D), where bleached versus healthy *Pocillopora acuta* were accurately predicted as bleached or healthy by linear discriminant statistical analysis. Each point represents a small biopsy sample taken from the corals, and multiple corals of the same genets were evaluated. Photos in A and B are courtesy of Dr. Andrew Bruckner, NOAA.

Mass spectroscopy, a workhorse for metabolomics, provides a useful tool for analyzing large amounts of data to identify metabolic biomarkers and has been successfully used in biomedicine to define disease states, including Alzheimer's disease, dementia, Parkinson's disease, and cardiovascular disease [57]. With this success, as well as decreasing costs and technological improvements, metabolomics is now applied for disease detection in coral models [48, 58, 59]. Direct analysis in real‐time mass spectrometry (DART‐MS), an emerging method for metabolomics that enables rapid metabolite fingerprinting of heterogeneous samples (e.g., plant tissues, living organisms, liquids) in an open environment [60], provides a promising approach for metabolic biomarker discovery in corals. The DART‐MS platform is also rapid, has minimal sample preparation requirements, and is cost‐effective after initial equipment acquisition. This approach has already been applied to corals to differentiate genera using their unique chemical signatures and, with optimization, has the potential to differentiate coral samples by species, clone identity, and bleaching status [61]. In terms of coral disease diagnosis, DART‐MS‐based approaches would facilitate metabolite biomarker discovery, differentiating disease states (e.g., black band disease, yellow band disease, stony coral tissue loss disease) by metabolic syndrome rather than pathogen (e.g., Figure 2). For example, a recent study demonstrated that coral disease states can be unobtrusively assessed by sampling coral mucus using fatty acid profiles to effectively differentiate healthy from diseased corals [62]. In cases of diseases that involve microbial consortia, this may provide a more reliable method for disease diagnosis than visual phenotyping and DNA approaches. After biomarker identification, diagnostic validation could proceed on the DART‐MS platform and be transitioned to colorimetric test strip assays for field‐deployable disease detection.

### 3.2 Monitoring Coral Spawning

Coral populations must be able to reproduce to recover from disturbances and sustain themselves over time. In recent years, researchers, practitioners, and managers have recognized the importance of sexually derived coral stock in expanding the genetic diversity and spatial scale of reef restoration efforts [6, 63, 64]. However, corals of the same species within a reef system may spawn on different nights or at different times of the night [65, 66, 67], preventing them from breeding with one another due to gamete degradation and/or dispersal. Synchronous spawning is critical to achieving fertilization [68, 69]. Evidence is mounting that worldwide, corals are spawning with increasing asynchrony, or not at all [70, 71, 72, 73], perhaps because anthropogenic changes such as ocean warming and light pollution disrupt their ability to reliably detect environmental cues. This phenomenon is known as **coral mass spawning asynchrony (CMSA**; see **Box)**. CMSA can lead to numerous downstream issues, such as inviable gametes, low fertilization success, and low genotypic mixing, all of which reduce the ability of populations to recover following disturbances and undermine ongoing conservation and restoration efforts. Particularly for endangered species, whose sparse numbers make them already subject to the “Allee effect”, which states that low population sizes can have a negative effect on individual fitness, and by extension, reproductive rate [74]. Therefore, the development of tools to assess reproductive status is a high priority for monitoring wild populations and to aid breeding programs.

Current approaches to study the coral reproductive cycle typically involve sampling for histology, which takes weeks to months to yield results, and “cracking” branches to visually assess the presence of gamete bundles, which is invasive and not well suited for massive (i.e., mounding) coral species or gonochores (separate male and female colonies). For species whose reproductive biology is less well‐known, metabolite and steroid hormone markers are a promising avenue for reproductive monitoring and interventions. Elevated concentrations of sex hormones have been described in corals during the annual spawning period [75], thereby supporting the idea that corals can endogenously synthesize vertebrate‐type sex hormones [76]. In addition, using preliminary data, Williams et al. [77] showed that thermal stress suppresses the levels of predicted coral sex hormones (validation of these metabolites is, however, still needed) when compared to ambient conditions, suggesting that CSMA may be detectable using hormone biomarkers. Next steps for steroid hormone biomarker discovery should include characterizing the annual coral steroid hormone cycle under both ambient conditions and stress across multiple species with distinct reproductive strategies. Biomarker validation should then be conducted under both controlled laboratory conditions and large‐scale field studies with wild populations. Finally, validated biomarkers can be integrated into prototype diagnostic tools, which may require private industry partnerships, and should be tested using the same samples to ensure assay precision and accuracy. In the future, the discovery of metabolite and steroid hormone markers may enable the application of POC tests for human diagnostics, such as pregnancy and fertilization, to facilitate reproductive monitoring and management. A detailed understanding of cyclical patterns of sex hormones across taxonomically diverse species that represent the breadth of reproductive strategies utilized by corals can potentially lead to strategies for the manipulation of coral reproduction using hormone therapy, increasing the capacity for and efficiency of various genetic and assisted reproduction interventions.

### 3.3 Thermal Stress Diagnosis and Prediction

To date, most work involving coral biomarkers has been conducted in the context of thermal stress, with a particular focus on transcriptomic signatures [78]. However, apart from CBASS [78], this research has yet to be widely translated into field‐deployable tools for thermal stress monitoring. Many of the field‐deployable tools currently used by restoration practitioners require visible assessment of bleaching for thermal stress diagnosis [15, 79–81]. However, molecular tools may be able to diagnose stress in corals before the onset of bleaching, allowing monitoring of bleaching thresholds in wild populations and rapid deployment of intervention measures for coral nurseries [82]. In addition, RNA may not be an ideal marker of coral health state given its short half‐life and high abundance variation across different species and within populations (for details, see Chille et al., [44]). Below, we describe two emerging biomarker‐based tools for thermal stress monitoring in corals: urinalysis test strips and lateral flow tests.

**FIGURE 3:**
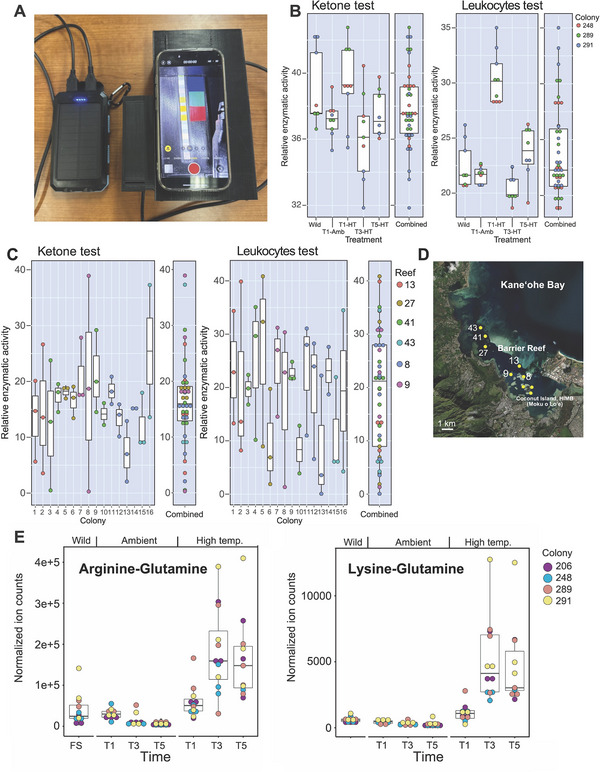
Analysis of coral metabolites. (A) Portable instrument for test strip analysis using a 3D printed smartphone holder. (B) Test strip results from analysis of ketone and leukocytes from thermal‐stressed colonies of *Montipora capitata*. The intraindividual variation is shown for three colonies (genotypes) that were either maintained in the natural environment (Wild) or treated in tank cultures with ambient (Amb) or thermal stress (HT) conditions over three time points. (C) Ketone and leukocytes test results from wild *M. capitata* colonies collected from six different reefs in Kāneʻohe Bay, Oʻahu, Hawaiʻi (D). Shown are standard box plots, with the boxes representing the first (Q1) to third (Q3) quartiles. The lines in the boxes are the median (Q2) values and “whiskers” extending beyond the boxes are the minimum and maximum values, excluding outliers. Figure reproduced from Meng et al. [84] under a Creative Commons Attribution 4.0 International License (http://creativecommons.org/licenses/by/4.0/). No modifications were made. (E) Accumulation of the dipeptides, arginine‐glutamine, and lysine‐glutamine under heat stress in nubbins from four *M. capitata* colonies (the same individuals from the same experiment shown in panel B). The abundance values are all significant (*p*‐value < 0.005) when comparing ambient T5 and heat stressed T5 using the Student's *t*‐test. Figure reproduced from Williams et al. [77] under a Creative Commons Attribution‐Non‐Commercial 4.0 License (https://creativecommons.org/licenses/by‐nc/4.0/). No modifications were made.

Urinalysis strips (Accutest URS 10; ca. $15‐25 for 100 strips) are used to detect metabolite biomarkers in humans that are diagnostic of metabolic syndromes (e.g., diabetes, kidney function, urinary tract infections) [83]. Recent work has shown that this tool is translatable in corals for thermal stress detection (Figure 3A) using a simple metabolite extraction protocol developed for coral nubbins that may potentially also be applicable to coral mucus [84]. Urinalysis test strips for thermal stress detection were validated at a population level in wild colonies of *Porites compressa* collected from six reefs in Kāneʻohe Bay, Oʻahu, Hawaiʻi (Figure 3D), as well as in controlled experimental systems with taxonomically distantly related species, including *Montipora capitata*, *Pocillopora acuta*, and *Porites compressa* (Figure 3B,C). Of the 10 diagnostic assays represented on these strips, two appear to be most applicable to corals: the ketone assay, which measures acetoacetate (a biomarker of glucose deprivation, elevated under stress conditions), and the leukocyte assay, which measures esterase activity (a biomarker of immune response, elevated under stress). The levels of these biomarkers vary across species based on their genotype and thermal resilience. Test strip results from *M. capitata* were compared to transcriptomic data generated from the same samples, which provided evidence supporting the stress syndromes detected by the strips [84]. To make the test strip assay more field‐deployable, a 3D‐printed smartphone holder was designed and paired with image processing software for rapid, large‐scale analysis of test strips (*TestStripDX*) (Figure 3A). This approach provides an example of how existing human POC tests can be readily adapted, with adjustments to sensitivity and detection range and limits, for coral health monitoring at the population level. *TestStripDX* serves as a model for developing additional colorimetric dipstick assays for thermal stress diagnosis and prediction. Next steps utilizing this approach should include pilot studies to validate the efficacy of this assay to diagnose stress across coral species and geographic regions, testing other human‐based colorimetric assays for thermal stress diagnosis or prediction, and discovery and validation of coral‐specific metabolic biomarkers that can be integrated into existing colorimetric assays.

**FIGURE 4:**
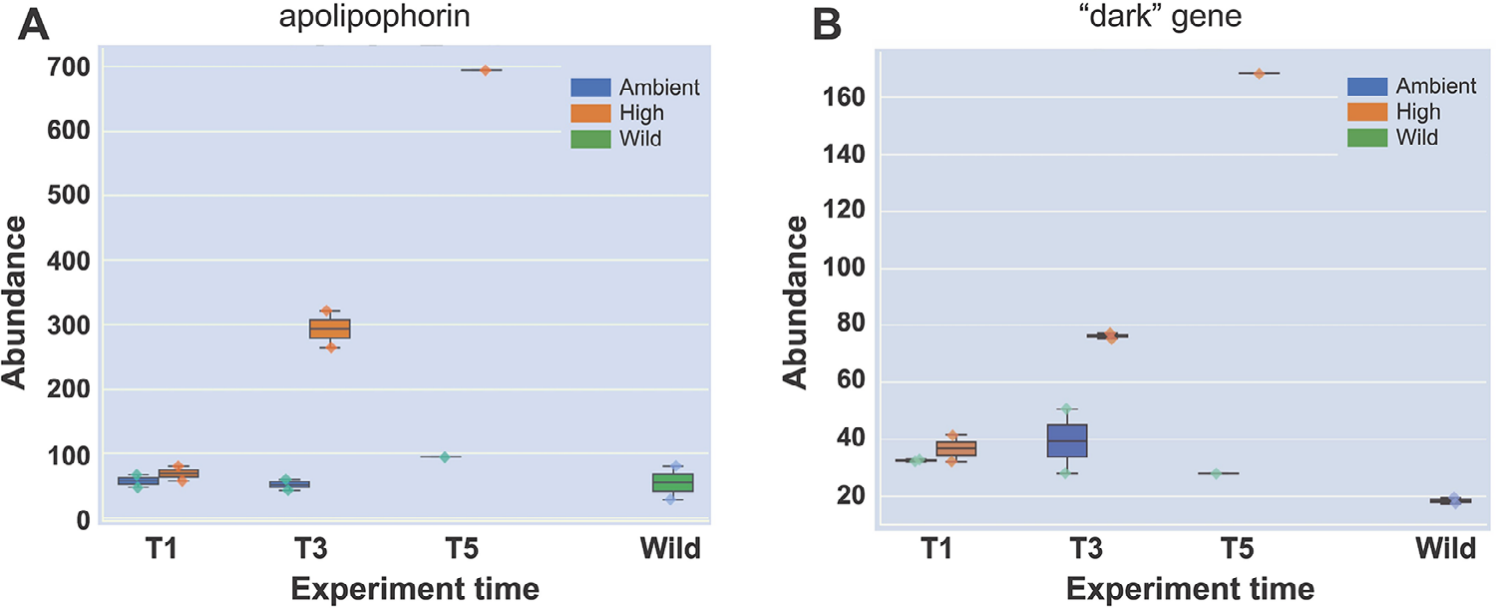
Protein abundance changes for *M. capitata* colonies maintained under ambient and thermal stress conditions. These are the same individuals from the experiment shown in Figure 2B. (A) Increase in apolipophorin abundance under thermal stress when compared to ambient conditions and for wild colonies. (B) Increase in a dark protein abundance under thermal stress when compared to ambient conditions and for wild colonies. Figure reproduced from Williams et al. [88] under a Creative Commons Attribution 4.0 International License (https://creativecommons.org/licenses/by/4.0/). No modifications were made.

Another promising approach for monitoring coral health in the field is the use of antibody‐based tools, such as lateral flow tests. Although metaproteomics research in corals is relatively new, with only a few studies exploring this rich omics data source [85, 86, 87], several protein‐based biomarkers show promise for integration into these cost‐effective, field‐deployable tools for thermal stress monitoring. Examples of coral proteins that show promise for integration into lateral flow tests or other antibody‐based tools include apolipophorin (Figure 4) [88], heat shock proteins 70 and 60, aB‐crystallin, ubiquitin, copper/zinc superoxide dismutase, and manganese superoxide dismutase. Symbiodiniaceae‐derived proteins include plastid small heat shock protein and homologs of the plant class I–IV small heat shock proteins [89]. Some unannotated “dark” proteins (proteins with unknown function) may also show promise for monitoring thermal stress. For example, Williams et al. [90] showed that a dark protein in *M. capitata* had an increase in abundance under thermal stress when compared to ambient conditions and wild colonies not undergoing bleaching. This protein has no significant hits to sequences in the nonredundant NCBI database, suggesting that it may have a novel function that is highly specific to corals. **Dark genes** (see **Box**) represent a rich source of targets (potentially comprising most proteins in the holobiont) that can be used for diagnostics and are potentially more informative of ecosystem health because they likely evolved in response to specific evolutionary constraints faced by the target organism. This makes dark genes potentially highly specific biomarkers of ecosystem health. The biological activity of a biomarker is irrelevant to its utility as a biomarker for diagnostic purposes pending it meets the needed sensitivity and specificity constraints to provide accurate assessment of coral health. As mentioned by Parkinson et al. [24], commercially available polyclonal antibodies are non‐specific to corals and vary batch‐by‐batch, making it difficult to standardize their use as a diagnostic. In addition, coral dark proteins do not have commercially available polyclonal or monoclonal antibodies for biomarker validation and assay development. Therefore, the advancement of protein‐based tools is contingent on the generation of new coral‐specific monoclonal antibodies (making lineage‐specific dark genes a promising target, for example, Stephens et al. [91]) or the validation of existing monoclonal antibodies targeting conserved protein targets. Future work toward the development of lateral flow assays should focus on field tests of protein biomarkers that have already been validated across diverse coral taxa in controlled lab settings, such as HSP60, which has been validated for its efficacy in diagnosing coral salinity, light, and temperature‐induced (heat and cold) stress [41, 92–96]. There is also an urgent need to generate a library of validated biomarkers that are predictive of stress or stress resilience prior to visible bleaching to aid in targeted intervention measures for coral nurseries during marine heatwaves or for targeting heat‐resistant colonies for coral gardening efforts. Despite these limitations, the response to the COVID‐19 pandemic has made it clear that antibody‐based POC testing platforms provide an invaluable tool for population‐level health assessment, which will greatly benefit the coral conservation field upon translation of the technology.

## 4 Implementation of Biomarker‐Based Tools

As coral restoration initiatives continue to increase the number of corals and species they work with and the ecological breadth and impact they wish to achieve, there is a growing need for rapid diagnostic tools that inform responsive management actions (i.e., early warning systems). As evident in the Caribbean during the 2023 mass coral bleaching and mortality event, large‐scale disturbances can potentially disrupt and derail coral restoration efforts and critical research on coral reef systems [97]. If tools for increasing the predictability of coral physiological responses during the critical periods of coral spawning and coral bleaching, such as those described herein, are integrated into conservation contexts, they will offer novel methods for responding to disturbances. By evaluating coral physiology in real‐time, restoration resources can be deployed more proactively, efficiently, and non‐invasively than with existing tools. Finally, the additional data collected by these tools can be incorporated into existing management and response plans, with population‐ or community‐level thresholds for novel metrics that trigger management responses and/or permit activities.

**FIGURE 5:**
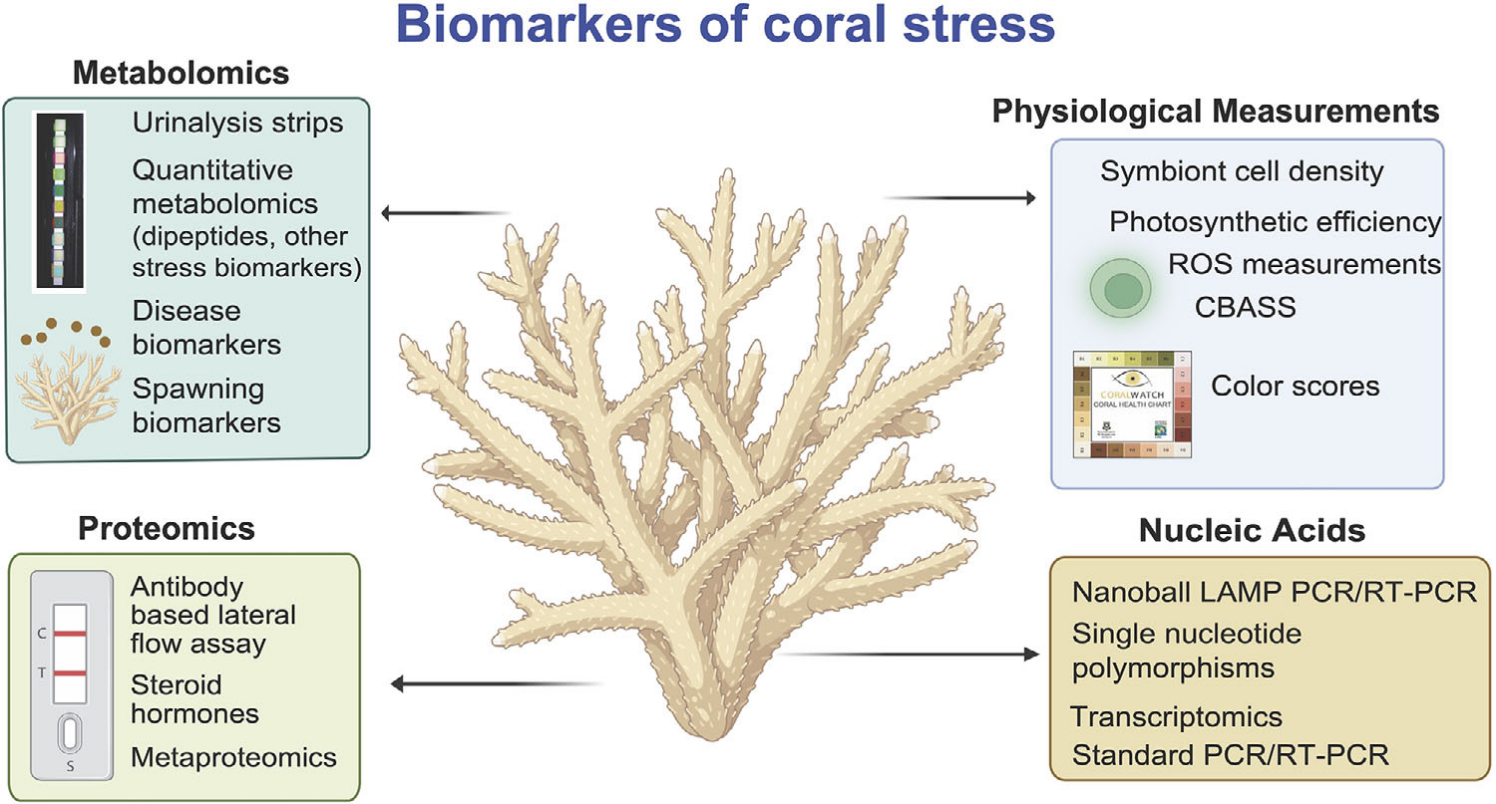
Summary of multi‐omics tools discussed in this review that show potential as biomarkers of coral health and disease. Figure created using Biorender.

To achieve the larger goals of informing and augmenting ongoing restoration efforts, these coral health toolkits should be shared as widely as possible with the restoration community, and validated, iterated, and adjusted based on their feedback (Figure 5). A broadly generalizable framework to guide biomarker discovery and validation has been proposed by Parkinson et al. [24]. Here, we provide guidance on the steps needed for large‐scale implementation. First, restoration practitioners representing a diversity of geographies and focal species should be trained on using these tools from initial sample collection through to data analysis and interpretation. Instruction should be hands‐on when possible, and dissemination can be supported or scaled using digital training modules, videos, and webinars. Trainees should be actively engaged to establish a suite of appropriate restoration responses given different potential results from the toolkit. Following training and pilot use in the field, feedback from practitioners should be gathered to validate toolkit utility and ensure products meet the needs of field practitioners. Given that many restoration efforts include active citizen science programs to aid with outplanting and monitoring restored corals, the toolkit should also be trialed for use within these programs to diversify the ways members of the public can engage in coral restoration. Feedback from both the citizen scientists and restoration practitioners should be integrated into future versions of tools, and proposals for new tools should be leveraged to improve the technology, training, and curricula. The ideal outcome would be for restoration programs worldwide to adopt regular use of these coral health tools to increase efficiency in their operations and engagement with citizen scientists and the public.

## 5 Conclusion

Approaches for coral health monitoring are rapidly developing and becoming more robust and multi‐faceted. Building these tools and planning their deployment following the example set by the biomedical field takes advantage of the vast experience and perspectives that exist in the field of human health. This will avoid reinventing existing POC testing platforms that need to be tested and applied to coral models. As described above, given the ancient derivation (i.e., compared to humans) and immense genetic diversity of coral hosts and algal symbionts, the challenge of coral health monitoring should be addressed in a stepwise manner. Limitations will become apparent throughout the process, such as the case of changing biomarker expression as corals adapt to stress and become resilient. As in the medical field, we must generate vast amounts of population‐ and community‐level data to address the complexities associated with coral genetic variation and how this interacts with other holobiont components. This aspect underlines the need to develop inexpensive and informative tools to heighten impact on coral conservation.

We propose a strategic, stepwise approach to develop and implement biomarker‐based POC tools effectively over the next decade:

- **Short‐term goals (∼1–5 years)**: Establish collaborations between researchers, restoration practitioners, local communities, private and governmental funding sources, and businesses; identify protein and metabolite biomarkers for disease and reproduction that can be integrated into existing POC technologies; and conduct large‐scale validation of existing thermal stress biomarkers.
- **Medium‐term goals (∼3–6 years)**: Conduct large‐scale field trials to validate disease and spawning biomarkers; determine the sensitivity and detection thresholds needed for each target population, given that this may vary between species and regions; and develop and test prototypes for thermal stress detection tools.
- **Long‐term goals (∼5–10 years)**: Finalize the development, validation, and implementation of validated POC prototypes, scale up deployment in conservation and restoration programs, and integrate biomarker‐based diagnostics into routine reef monitoring strategies.

Ultimately, innovation in coral health diagnostics must stem from collaborations between funders, researchers, restoration practitioners, businesses, and communities. These partnerships will be critical to bridging the financial and logistical gaps that need to be overcome to support reef restoration efforts and protect these precious resources for future generations.

## Author Contributions

D.B., E.E.C., and T.G.S. contributed equally to manuscript conceptualization and formulation of the overarching goals of the review. D.B. secured funding to support this work. E.E.C. conducted the literature review. S.N., D.B., and M.G. contributed to visualization and data presentation. E.E.C. and D.B. wrote the original draft with help from T.G.S., M.G., O.M.W., P.M.M., and A.N. All authors participated in editing and revising the original draft. E.E.C., D.B., and T.G.S. revised and edited the final manuscript. All authors reviewed and approved of the final work.

## Conflicts of Interest

E.E.C., T.G.S., S.N., and D.B. disclose their involvement in the startup OceanOmics Inc. that has utilized some of the data and insights described in this manuscript.

## Supporting information

Supporting Information

## Data Availability

The datasets that support the findings of this study are publicly available. No unique datasets were generated for this review article.
