# Supplementary material for: Coral Restoration in the Omics Era: Development of Point‐of‐Care Tools for Monitoring Disease, Reproduction, and Thermal Stress
Source: Bioessays. 2025 Apr 26;47(6):e70007. doi: 10.1002/bies.70007 (PMC12101048; doi:10.1002/bies.70007)
Supplement: Supplementary file 1 — Supporting Information [file BIES-47-e70007-s001.docx]

**Supporting Information**

**Towards development of point-of-care diagnostic tools for coral health monitoring: Enhancing restoration efforts in the omics era**

**List of authors:**

Erin E. Chille^1,*^, Timothy G. Stephens^1^, Shrinivas Nandi^1^, Haoyu Jiang^1^, Michael Gerdes^2^, Olivia M. Williamson^3^, Alexander Neufeld^4^, Phanor Montoya-Maya^4^, Debashish Bhattacharya^1,*^

**Author affiliations:**

^1^Department of Biochemistry and Microbiology, Rutgers University, New Brunswick, New Jersey, 08901, USA

^2^CapitalCorals INC, Albany, New York, 12206, USA

^3^Revive & Restore, Sausalito, California, 94965, USA

^4^Coral Restoration Foundation^™^, Overseas Hwy, Tavernier, Florida, 33070, USA

^*^Corresponding author

**Abbreviations:**

whole genome duplication (WGD)

**Support for the development of diagnostic tools targeting *Symbiodiniaceae***

Support for this idea comes from analysis of the genomes of two *Durusdinium trenchii* strains (*D. trenchii* CCMP2556, 1.71 Gbp genome size and *D. trenchii* SCF082, 1.64 Gbp genome size). This genus of facultative symbionts is renowned for conferring thermal stress tolerance to coral hosts,^[1,2]^ leading to its spread throughout the Caribbean.^[3]^ The draft genome assemblies revealed several interesting features. Most important, the common ancestor of these strains underwent a whole genome duplication (WGD) that led to the fixation of large families of ohnologs (i.e., gene duplicates derived from WGD). These ohnologs offer the opportunity to study gene expression patterns to elucidate how selection may have shaped their functional differentiation post-WGD. The experiments done by Dougan et al.,^[4]^ were underpinned by the hypothesis that genome large-scale growth (i.e., WGD) most likely occurred during the free-living phase of *Durusdiniun*, because the opposite, genome reduction, is the expected outcome of long-term symbiosis.^[5]^ Analysis of differential gene expression of strain CCMP2556 was done using free-living cultures and cells in symbiosis with the anemone *Exaiptasia pallida* under ambient (28°C) and thermal stress (34°C) conditions. These results revealed that most ohnolog pairs showed differential gene expression, many under the two different lifestyles. This suggests that the dual lifestyle is a major driver of post-WGD gene expression with fluctuating environmental conditions (e.g., nutrient availability, thermal stress) during the free-living stage likely driving fixation of ohnologs. These novel traits made *Durusdinium* a better symbiont, able to maintain a stable host-symbiont relationship (e.g., *via* efficient nutrient/metabolite exchange) under stressful conditions for the coral holobiont.^[4]^ These ground-breaking results demonstrate further why we need to consider symbionts in coral conservation plans as major players in algal and coral evolution that extends far beyond simply provisioning photosynthates to cnidarians as so-called “zooxanthellae”.

**References**

1. Rowan, R. (2004). Coral bleaching: thermal adaptation in reef coral symbionts. *Nature*, *430*(7001), 742.

2. Berkelmans, R., & van Oppen, M. J. H. (2006). The role of zooxanthellae in the thermal tolerance of corals: a “nugget of hope” for coral reefs in an era of climate change. *Proceedings. Biological Sciences*, *273*(1599), 2305–2312.

3. Pettay, D. T., Wham, D. C., Smith, R. T., Iglesias-Prieto, R., & LaJeunesse, T. C. (2015). Microbial invasion of the Caribbean by an Indo-Pacific coral zooxanthella. *Proceedings of the National Academy of Sciences of the United States of America*, *112*(24), 7513–7518.

4. Dougan, K. E., Bellantuono, A. J., Kahlke, T., Abbriano, R. M., Chen, Y., Shah, S., Granados-Cifuentes, C., van Oppen, M. J. H., Bhattacharya, D., Suggett, D. J., Rodriguez-Lanetty, M., & Chan, C. X. (2024). Whole-genome duplication in an algal symbiont bolsters coral heat tolerance. *Science Advances*, *10*(29), eadn2218.

5. González-Pech, R. A., Bhattacharya, D., Ragan, M. A., & Chan, C. X. (2019). Genome Evolution of Coral Reef Symbionts as Intracellular Residents. *Trends in Ecology & Evolution*, 1–8.
